# Discovering Tumor Suppressor Genes Through Genome-Wide Copy Number Analysis

**DOI:** 10.2174/138920210791616734

**Published:** 2010-08

**Authors:** S. Michael Rothenberg, Jeff Settleman

**Affiliations:** Massachusetts General Hospital Cancer Center and Harvard Medical School, 149, 13th Street, Charlestown, MA 02129, USA

**Keywords:** Array CGH, copy number analysis, cancer, tumor suppressor genes.

## Abstract

Classical tumor suppressor gene discovery has largely involved linkage analysis and loss-of-heterozygosity (LOH) screens, followed by detailed mapping of relatively large chromosomal regions. Subsequent efforts made use of genome-wide PCR-based methods to detect rare homozygous deletions. More recently, high-resolution genomic arrays have been applied to cancer gene discovery. However, accurate characterization of regions of genomic loss is particularly challenging due to sample heterogeneity, the small size of deleted regions and the high frequency of germline copy number polymorphisms. Here, we review the application of genome-wide copy number analysis to the specific problem of identifying tumor suppressor genes.

## INTRODUCTION

Ever since the seminal demonstration by Bishop and Varmus more than 30 years ago that transforming oncogenes arise from normal cellular genes, the discovery of additional cancer-specific mutations has fueled the hope of diagnosing and treating cancer with tumor-specific modalities [1]. However, although identifying the driving genetic lesions for select cancers has led to improved diagnostics and therapies in some cases, the total number of patients who benefit from these approaches remains a relatively small fraction of all patients suffering from cancer [2-7]. To identify additional cancer genes, a variety of unbiased, genome-wide approaches have emerged that can detect a large fraction of the genetic differences between human tumors and their normal tissue counterparts. Among these, the development of array-based genomic hybridization technologies has paved the way for interrogating an ever increasing density of probes for markers distributed across the cancer genome for increases and decreases in intensity suggestive of somatic alterations causally related to tumor formation. To facilitate the analysis of the very large datasets generated by such methodologies, several automated approaches have been developed to systematically refine the number of candidate cancer genes for further genetic and functional characterization. These have led to the nomination of numerous candidates whose biological significance requires deeper genetic analysis and robust functional validation. Here, we review the application of array-based gene copy number analysis to the specific problem of identifying tumor suppressor genes. We start by reviewing the discovery of *bona fide *tumor suppressor genes for common themes that may guide the interpretation of candidates identified through genomic approaches. Next, we review the development of genome-wide approaches aimed at the global identification of copy number alterations in cancer. We then focus on array-based copy number analysis and review the major steps in converting probe-level intensity measurements to deleted regions harboring candidate tumor suppressor genes. Finally, we discuss specific examples of new candidate tumor suppressor genes identified through genome-wide copy number analysis and we summarize the evidence for their involvement in human cancer.

## DISCOVERING *BONA FIDE* TUMOR SUPPRESSOR GENES

Historically, genetic approaches for discovering tumor suppressor genes (TSGs) have required validating several candidate genes contained within a large chromosomal region implicated by linkage analysis in families afflicted with cancer predisposition syndromes or targeted by tumor-specific allelic loss (so called loss-of-heterozygosity/LOH). Several common themes have emerged from these efforts (Table **1**).

### 1. Rare Cases Associated with Focal (Homozygous) Deletions can be Informative

In the “pre-genomic” era, the chromosomal regions implicated in cancer by linkage analysis or LOH screens were usually very large (on the order of many megabases), and would therefore normally require many iterative steps of genotyping with many more genetic markers, and eventually, the sequencing of many candidate genes to detect inactivating mutations indicative of the targeted TSG. In most cases, uncovering the targeted gene relied on the discovery of rare cases with focal deletions (germline heterozygous deletions for genes predisposing to cancer and somatic tumor-specific homozygous deletions for sporadic tumors) encompassing one or a few genes (Table **1**). In fact, the recognition of the value of tumor-specific focal homozygous deletions to implicate new TSGs provided much of the impetus for the development of unbiased, genome-wide approaches for detecting them (see discussion below) [8-11].

### 2. Very Small Deletions can Inactivate Genes

In theory, deletion of a single coding nucleotide will cause a frameshift mutation that can lead to gene inactivation through protein truncation. Although detecting single nucleotide deletions essentially requires single nucleotide sequencing, very small deletions encompassing one or a few genes have been instrumental in cloning many important TSGs (Table **1**) [12-22]. Identifying the *APC *gene relied on an analysis of two cases with focal deletions spanning just three genes [14,16]. Focal homozygous deletions in cancer cell lines were instrumental in the identification of *INK4A/B* as one of the most common targets of genetic inactivation in human cancers [17,22]. Loss of heterozygosity affecting nearly the entire long arm of chromosome 10 is extremely common in primary gliomas, and the identification of *PTEN *was made possible by the discovery of rare cell lines with intragenic homozygous deletions affecting *PTEN *and no other flanking genes [18,20]. A deletion of just 25 nucleotides (spanning an exon-intron boundary and leading to aberrant splicing) was critical to implicating *WT1 *in Wilms’ tumor [12,15]. As can be seen in Fig. (**1**), a high resolution scan of genomic loci known to be targeted by homozygous deletions reveals both broad deletions encompassing several genes and very focal, intragenic deletions disrupting just one or a few coding exons and therefore directly implicating the individual, targeted gene. 

### 3. Detecting Homozygous Deletions is Extremely Sensitive to Background Effects and Stromal Admixture

Each of the examples described above relied on the presence of the deletion in every cell within the analyzed samples, either because the deletion was present in the germline (e.g. for cancer predisposing TSGs) [12,14-17] or because cancer cell lines or primary cultures (presumably, clonally-derived) were utilized (for somatically inactivated TSGs) [17,20]. For both PCR-based approaches and, as discussed below, array-based copy number analyses, the detection of deletions may be particularly confounded by the presence of mixed tumor-associated stroma, since the presence of non-cancer diploid genomes can lead to preferential detection of the non-deleted, normal DNA [8,23]. In addition, accurately measuring genomic losses is inherently more difficult than detecting amplifications, since losses are limited to only one or two copies, and any residual signal will be extremely close to the lower limits of detection of the platform being utilized.

Each of these features may significantly impact the discovery of candidate TSGs by genome-wide copy number analysis. Small deletions consisting of one or a few genomic probes with hybridization signals barely above background may easily be obscured within the significant noise associated with the simultaneous measurement of literally hundreds of thousands of genetic markers. The invariable presence of stromal cells in primary tumor specimens may further decrease the ability to detect discrete losses indicative of novel TSGs. Therefore, the specific consideration of background effects and stromal admixture by individual genomic platforms and computational algorithms is critical for establishing candidate TSGs proposed on the basis of genome-wide copy number analyses. The sensitivity of deletions to these confounding effects may be contrasted with chromosomal regions of copy number gain/amplification that may harbor novel oncogenes. Unlike inactivating deletions, gains that may lead to oncogene activation through increased expression must extend at least the entire length of the targeted gene (and in practice often span several genes). In addition, the degree of amplification may be many times the normal diploid copy number and therefore will be less subject to the confounding effects of background signal and admixed stroma. 

## IDENTIFYING TUMOR SUPPRESSOR GENES BY GENOME-WIDE COPY NUMBER ANALYSIS

### PCR-Based Approaches

Initial unbiased efforts to screen the cancer genome for regions of genomic loss that potentially harbor tumor suppressor genes utilized PCR to enrich the DNA sequences present in normal genomic DNA that were absent from tumor DNA. Among several methods developed to identify the differences between two complex genomes, representation differentiation analysis (RDA) has been most successfully used to identify novel TSGs [8,9]. The RDA methodology consists of restriction digestion, subtractive hybridization and selective PCR amplification to generate simplified “representations” of the genome using mixed tumor and matched normal samples. RDA is extremely powerful for detecting rare differences in largely identical genomes (e.g. viral sequences, tumor samples with matched normal DNA available). RDA was instrumental in the cloning of *PTEN, BRCA2 *and other candidate tumor suppressor genes [18,24,25]. However, several features have complicated its use as a screening tool for cancer gene identification: (1) technical complexity (which limits the number of samples that can be analyzed); (2) genomic instability in solid tumors, leading to the detection of many candidate regions that appear to be decreased in tumor DNA (compared to normal); (3) copy number polymorphisms, which, in the absence of matched normal tissue DNA, lead to many false positives; and (4) the fact that candidate regions are identified *en masse*, and must be subsequently deconvoluted to permit validation of individual loci. Nevertheless, several improvements have been made to increase the power of RDA. Using tumor genomic DNA from genetically engineered mouse models and strain-matched normal DNA can decrease the impact of germline copy number variation, while combining the initial subtraction and amplification steps with array-based hybridization permits rapid deconvolution of complex mixtures of differentially amplified loci (representational oligonucleotide array analysis/ROMA—see below) [25,26]. 

## MICROARRAY-BASED COMPARATIVE GENOMIC HYBRIDIZATION (CGH)

The initial demonstration of tumor-specific genomic imbalance by traditional metaphase karyotyping has led to increasingly sensitive methods for detecting cytogenetic abnormalities in cancer. With comparative genomic hybridization (CGH), differentially fluorophore-labelled tumor (e.g. fluorescein) and normal (e.g. rhodamine) DNA are co-hybridized to normal metaphase spreads in the presence of unlabelled Cot-1 DNA (to block off-target binding to repetitive sequences), permitting quantitative detection of discrete regions of chromosomal gain or loss (as determined by the ratio of fluorescein-to-rhodamine fluorescence) [27]. CGH has permitted the cataloguing of human tumors by recurrent regions of gains and losses that correlate with clinical features and have helped to pinpoint causative cancer genes [28]. However, the use of metaphase chromosomes to localize genomic alterations and the high complexity of the probes limit its detection resolution to ~20Mb. A related methodology, spectral karyotyping (SKY), combines multicolor fluorescence in situ hybridization with spectral analysis that has been particularly valuable for detecting chromosomal translocations, though still with limited resolution (1-2Mb) [28].

The development of DNA microarrays, initially for mRNA expression analysis, provided two significant advantages over traditional CGH: (1) increased resolution, limited only by the density of genomic probes on the array (expected to increase further with the completion of the human genome sequencing project) [29,30]; and (2) highly parallel analysis, permitting the identification of individual candidate regions (for subsequent validation) in a single step. Initial applications of array-based CGH used large insert genomic DNA (e.g. BAC) clones that provided higher resolution (~40kB) than traditional CGH and the quantitative measurement of DNA gains and losses. However, increasing the resolution further was limited by probe complexity as well as the chemistry used to couple the genomic probes to the array surface, such that smaller probes were not bound effectively [10,31,32]. The development of complementary DNA (cDNA) microarrays and their application to the determination of DNA copy-number variation in human tumor samples represented a significant advance in genomic analysis of tumors by providing the first genome-wide view of copy number alterations at gene resolution [11,30]. In addition, the demonstrated ability to robustly detect single and two-copy losses (despite the much greater complexity of genomic DNA compared to cellular mRNA) is critical to detecting tumor suppressor genes, since genomic losses are discrete and limited in amplitude (e.g. loss of one or both alleles) compared to gains [11]. Another important advantage of cDNA microarray-based CGH is the ability to characterize both copy number and gene expression patterns in parallel in the same samples and on the same array platform, enabling candidate genes to be narrowed to those whose genomic gain or loss is associated with corresponding changes in gene expression (and thus are the likely targets of the alteration).

Arrays of oligonucleotide probes were developed in parallel to cDNA arrays [26]. The major platforms in current use consist of arrayed probes of 60-70 nucleotides for CGH (e.g. Agilent); or 25 nucleotide probes (e.g. Affymetrix), on which tumor and reference hybridizations are carried out separately, with copy number inferred indirectly by comparison. In addition to determining copy number, Affymetrix arrays are designed to detect single nucleotide polymorphisms (SNPs), and therefore, can be used for simultaneous determination of copy number and LOH at every analyzed locus from a single hybridization, even in the absence of a matched normal sample [23,33]. This may permit determination of the actual genetic mechanism underlying LOH events (e.g. copy-reducing from copy-neutral), and could reveal additional samples for deeper sequencing to identify inactivating mutations (e.g. point mutations) not apparent from copy number analysis. An important feature of most oligonucleotide probe-based methodologies is the requirement that the complexities of the analyzed genomes be significantly reduced (by restriction digest and PCR) to permit accurate hybridization. These approaches may therefore be quite sensitive to SNPs (that prevent restriction enzyme cleavage) and PCR artifacts that can contribute to misidentification of altered loci. Despite these potential limitations, oligonucleotide arrays provide increasingly high resolution and are capable of detecting alterations located within intergenic and nongenic regions of the genome, and have therefore become the preferred platform for gene expression and copy number analysis. 

Additional methodologies for copy number analysis include bead-based oligonucleotide arrays (e.g. Illumina) and digital karyotyping [34]. The former is very similar to arrays of chemically immobilized oligonucleotides but uses silica bead-based capture technology with more robust and uniform display of probe oligonucleotides, and therefore yields more accurate and complete measurement of probe hybridization (and furthermore does not require PCR-based representation generation or pre-labeling of genomic DNA). The latter approach, which utilizes combinations of restriction enzymes and PCR to generate 21-bp tags from genomic DNA that are concatamerized and sequenced to determine location and tag density and then computationally mapped, has been used to isolate candidate cancer genes, but may be limited in scope by the requirement for labor-intensive deep sequencing and decreased mapping resolution [35]. Finally, the feasibility of using single-molecule sequencing for identifying focal gains and losses based on density of reads has recently been demonstrated [36], although, like digital karyotyping, given cost and labor, is currently limited to analysis of only a few samples in any given study. A summary of the major array copy number analysis platforms is provided in Fig. (**2**).

## AUTOMATED DISCOVERY OF AMPLIFICATIONS AND DELETIONS

An explosion in the generation of array-based copy number data sets has led to an increasing number of computational approaches for deriving information about candidate cancer genes from probe-level data. The following section reviews the major steps in converting probe-level intensity data into regions of copy number alteration that may harbor cancer genes. 

### Converting Hybridization Intensity to Copy Number

Methods for converting raw probe intensity data to copy number information depend on the microarray platform (e.g. cDNA or oligonucleotide, CGH versus independent hybridization of controls). In general, following quality control and correction for background array noise, the hybridization signal from each probe on the array is normalized to the corresponding signal from a known or inferred diploid genome(s). SNP arrays provide probe-specific estimates of non-specific hybridization by incorporating single nucleotide mismatched probes (for each matched SNP probe) that permit probe-specific estimation of non-specific hybridization (copy number is determined using model-based estimates that account for the signal from both matched and mismatched probes [37]). To permit more robust comparison between separately arrayed samples (that may be subjected to systematic, array-specific differences in hybridization conditions), the hybridization intensity and/or normalized copy number for each probe in each sample is usually “centered” to the median value of all or select (e.g. “invariant”) probes on the particular array (arrays processed in the same batch may also be centered to the array with the median overall intensity among the arrays in the batch).

The choice of reference samples can significantly impact the accuracy of copy number measurements. The use of a single matched (e.g. from the same patient) reference sample to normalize each tumor sample may reduce the frequency of false-positives by controlling for germline copy number variations (see below); however, variable sample quality can make comparisons among different tumor samples more difficult [38]. Therefore, even when matched reference samples are available, a single reference consisting of multiple normal samples is often used. In the case of array CGH, these may be pooled prior to labeling and cohybridization (to each tumor sample). For SNP arrays, the “normals” are arrayed individually and then pooled computationally, most commonly by normalizing each sample probe to the average single intensity of that probe in the reference samples. Some authors have recommended the use of a standardized collection of normal reference samples, the polymorphic features of which have been extensively characterized (e.g., derived from the HapMap Project) [38]. 

### Converting Probe-Level Signals to Genomic Copy Number

Although in theory the copy number at each genetic locus should be defined by integer values (e.g. single copy loss, homozygous deletion or gain), intermediate values are more typically observed in array analyses. Sources of “noisy” copy number measurements include polymorphisms present in the arrayed DNA, probe sequence-specific variability in hybridization kinetics, incorrectly mapped probe locations and stromal admixture. A variety of computational methods have been developed to “automatically” delineate genomic regions of gains from probe-level data [26,39-49]. Although the mathematics differs among the individual methods, the general approach is: (1) sample by sample and chromosome by chromosome, combine data from neighboring markers with the same underlying copy number; (2) identify statistically significant transitions in copy number for neighboring probes using a reference distribution from normal tissue hybridizations or through permutation testing; and (3) repeat iteratively until significance is maximized for each copy number transition, producing genome “segments” consisting of variable numbers of contiguous probes with statistically similar copy number. Segmentation algorithms are essential for simplifying large datasets consisting of many samples to a manageable number of discrete alterations for subsequent analysis. However, to identify tumor suppressor genes, it is important to consider how each algorithm handles two features of variability in probe-level data, smoothing and small segments.

### Smoothing

Smoothing is the process in which each segment is assigned a copy number equal to the average of all of the probes it contains. Smoothing may reduce “noise” in the data by reducing the appearance of underlying probe-to-probe variability in hybridization, thereby increasing sensitivity and specificity [11]. However, smoothing may obscure precise detection of alteration boundaries and very small homozygous deletions comprised of one or a few probes, particularly when a homozygous deletion arises from one large-scale and one focal allelic loss (Fig. **3A**). 

### Small Segments

**Fig. (1):**
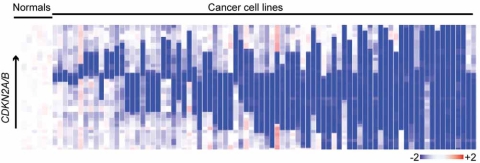
Homozygous deletions of *CDKN2A/B* in human cancer cell lines. Heatmap of log[2] copy number demonstrates targeting of this locus by both broad homozygous deletions in blue (red indicates gains) affecting neighboring genes (rightmost samples) and intragenic homozygous deletions (leftmost samples) that remove individual coding exons without impacting neighboring genes. This figure was adapted from [85].

The problem of individual outlier probes was initially appreciated from the comparison of normal-normal hybridizations using oligonucleotide array-based CGH, in which many stand-alone probes appear as minor losses and gains, manifesting as a “shell” of probes appearing primarily as many single-copy losses and gains [26]. These outlier probes consist of false-positive aberrations--hybridization artifacts, incorrectly mapped probes, SNPs that create or destroy sites for cleavage by the enzyme used to fragment the genomic DNA before hybridization—and potentially true positive, focal losses (and gains). Most algorithms employ methods to filter out potential outliers *via *smoothing, “fusion” to the closest large aberration, or by simply ignoring segments comprised of less than a pre-specified number of probes (usually 5-8) [26,50-53]. However, depending on the density of probes, true copy number losses comprising one or a few probes could in fact span many kilobases, and therefore inactivate the affected gene (Fig. **1**). 

### Scoring an Altered Region as Deleted

Scoring of losses requires a careful consideration of alteration amplitude. Most approaches pre-specify fixed copy number thresholds, either empirically or based on the distribution of probe intensities in reference samples; probes or segments with copy numbers falling outside of the threshold are considered significantly altered [51,52,54]. Some investigators apply multiple thresholds to distinguish low-level gains from high-level amplification and single copy losses from homozygous deletions [38,50]. 

Scoring deleted regions with such fixed thresholds is made particularly challenging by the inherent difficulty in accurately measuring the true signal (above background) from low intensity hybridizations. Although a deletion should result in 50% (heterozygous) or total (homozygous) loss of signal from the resident probes, even for homogeneous tumor cell populations (e.g. cell lines), significant probe-to-probe variation in copy number is observed across known genomic deletions, a result of several factors, including noisy measurements at the lower end of the dynamic range, smoothing, tumor heterogeneity, allele-specific copy number changes and negative skew in the distribution of copy number around the diploid peak [38]. Accurate scoring of deletions may be particularly confounded by the presence of contaminating stromal DNA in primary tumor specimens. Although most approaches exclude samples with < ~70% tumor cells, scoring may vary between pathologists and may not account for polyclonal tumor cell populations or extensively admixed stroma. The consequences of stromal admixture have been extensively characterized in “mixing experiments”; the data demonstrate that detection of homozygous deletions is extremely dependent on tumor purity [23]. Methods to minimize the effect of stromal admixture include tissue micro-dissection or the use of flow cytometry to separate aneuploid from diploid cells in patient samples [26,55]. 

### Germline Copy Number Variants

**Fig. (3):**
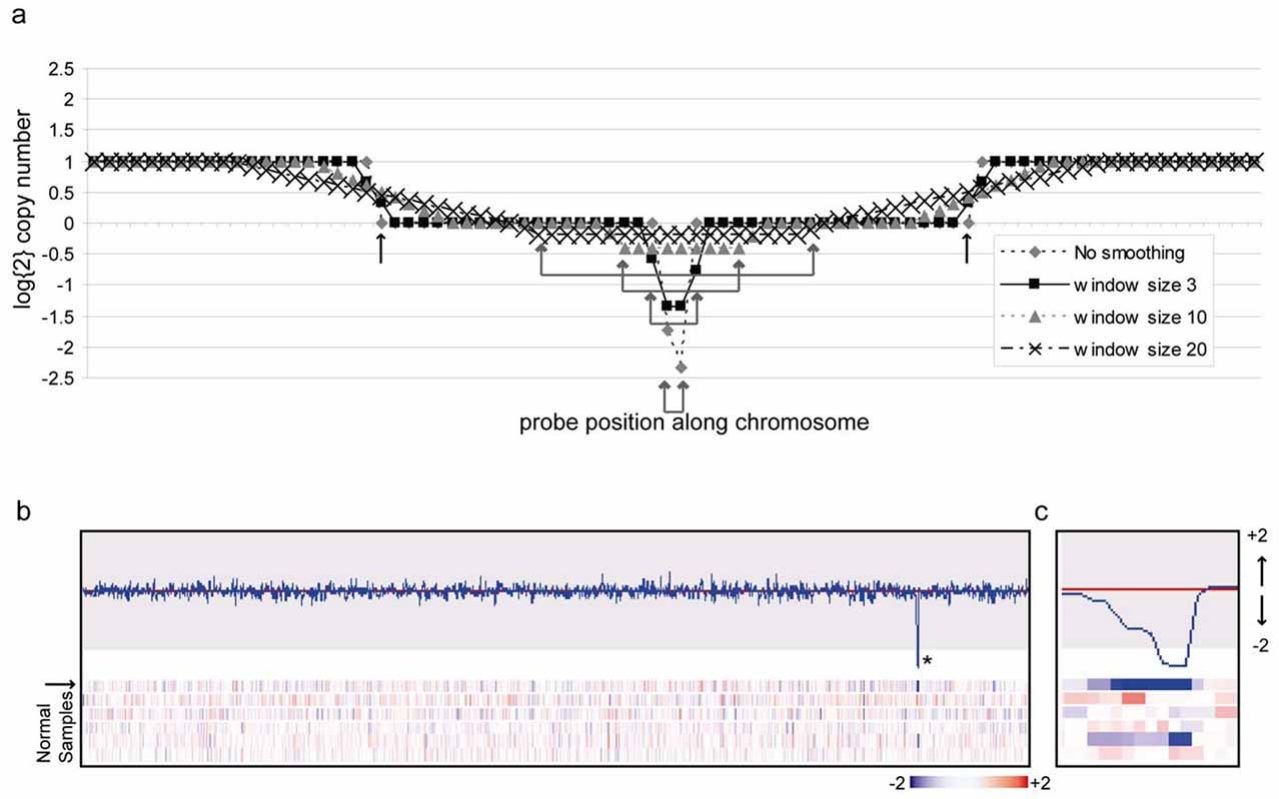
Detecting focal losses by copy number analysis. (**a**) The effect of median smoothing of probe copy number is demonstrated for atheoretical locus targeted by a focal homozygous deletion comprised of overlapping large (boundaries indicated with black arrows) and focal(paired gray arrows) alleic losses. As the probe window size for smoothing is increased, the focal deletion becomes obscured by the signalfrom surrounding probes. (**b**) Heatmap and graphical display of log{2} copy number for normal tissues. Genomic DNA from six normalsamples (peripheral blood mononuclear cells) was hybridized to Affymetrix Human Mapping 250K Sty Arrays and analyzed with dCHIP software (http://www.hsph.harvard.edu/~cli/complab/dchip/). Shown is a portion of chromosome 19 demonstrating a focal loss spanningseveral probes (asterisk) in one sample (indicated by black arrow), consistent with a germline copy number polymorphism. (**c**) higher resolutionview of region indicated by asterisk.

Germline genomic variation, associated primarily with short (0.1-1kB) insertions and deletions (Indels) and longer (>1kB) gains and losses (copy number variants/CNVs), has been demonstrated in ~12% of the genome, at frequencies as high as 10% of the normal population, and these often involve gene coding sequences (Fig. **3B**) [26,55]. A moderate correlation between CNV regions and segmental duplications has led to the suggestion that they may occur in regions of the genome prone to recombination events (and therefore could arise *de novo*). In addition, a recent study suggests that the majority of the copy number differences between any two individuals arise from a limited set of common, inherited polymorphisms [56]. Although germline copy number variations have been demonstrated to contribute to disease susceptibility, the contribution of individual polymorphisms to any particular phenotype is (so far) small [57-59]. Whether germline copy number losses predispose to tumor formation is not yet known, although, as for other diseases, the overall contribution of any individual variant to tumorigenesis may be small.

CNVs present a unique challenge to accurately identifying tumor suppressor loci because a germline deletion detected in a tumor by copy number analysis may be incorrectly considered to be of somatic origin. Furthermore, depending on the background rate of generation of genomic losses, a pre-existing heterozygous copy number loss could be converted to a homozygous deletion through somatic loss of the other allele (yet this still may represent a passenger mutation). Homozygous deletions overlapping pre-existing heterozygous CNVs have been observed, although the frequency at which this occurs has not been systematically determined [26]. 

Normalizing each tumor sample to matched normal tissue can help to establish the germline or somatic origin of each tumor alteration, but is impractical for the reasons discussed above. Therefore, most studies manage the potentially confounding influence of common CNVs by eliminating from consideration any altered probes that map to known CNVs, or by excluding entirely genes overlapping with frequent CNVs, based on the growing database of described genomic variants [55]. Nevertheless, many candidate tumor suppressor genes proposed on the basis of copy number analyses are also marked by frequent germline variation (Table **2**); this may complicate determining the true contribution to tumor formation (see discussion of individual candidate genes below). 

### Estimating the Background Rate of Aberration

Early attempts to define altered genomic regions potentially harboring novel cancer genes prioritized regions with recurrent alteration—those detected in more than one sample [23,40,60]. However, depending on underlying mutation rates and the number of samples analyzed, even recurrent alterations could represent byproducts of tumor genome instability rather than true driver aberrations. Therefore, as with determining the underlying rate of point mutations in tumor genome re-sequencing studies, more recent copy number analyses have attempted to estimate the background rate of mutation as the basis for determining statistical significance. Estimating the rate of point mutations is most often achieved by determining the rate of silent mutations within coding sequences and/or point mutations in non-gene containing regions of the genome (these are presumed to be phenotypically neutral and therefore not acted upon by selection) [61-65]. Furthermore, the wealth of recently generated tumor resequencing data has permitted the influence of nucleotide context on the rates of mutation to be incorporated into models of the background mutation rate. By contrast, the phenotypic consequences of alteration location, size, amplitude and sequence are less well understood. Two recent studies described genome features of human cancer cell lines associated with a higher rate of homozygous deletions (which also varied depending on the tissue of origin) [66,67]. This could suggest that many recurrent homozygous deletions detected in human cancer genomes lie within fragile sites prone to genomic losses rather than marking true cancer genes, thus complicating the identification of *bona fide *tumor suppressor genes on the basis of genome-wide deletion analysis.

Given this uncertainty, the most common strategy is to approximate the background rate (by binomial distribution or permutation testing) with the overall rate of alterations, assuming random distribution across the genome [38,51,53,68,69]. Most approaches are inherently conservative, e.g. they overestimate the background rate because they include alterations affecting *bona fide *cancer genes (which are likely true driving alterations rather than nonspecific passengers). Consequently, this approach could fail to detect true driver aberrations that occur at relatively lower frequency.

### Considering the Degree of Copy Number Change

Some approaches incorporate alteration amplitude (how many copies of a gene are gained or lost) into the determination of significance (e.g. together with the frequency of the observed alteration). The general approach is to set very low thresholds for initial scoring of gains and losses, followed by weighting of each alteration by the actual amplitude. Such weighting may be carried out using a continuous scale, or with discrete classes (e.g. single-copy gain, single-copy loss, amplification, or homozygous deletion) [38,50,51]. For amplifications, this may be useful; thus, the dynamic range of gains extends from single copy to more than 30, and, in general, the degree of amplification correlates with overexpression at the protein level (and thus the degree of activation of the targeted gene). However, for deletions, there are only two amplitudes: loss of one or both copies (what is the real meaning of commonly observed noninteger copy number values, and how should they be weighted?) Therefore, depending on the relative contribution of alteration frequency versus amplitude to the overall score, the true importance of particular genomic losses could potentially be under- or over-estimated.

### Finding the Individual Gene(s) Targeted by a Deletion

Although validating candidate genes targeted by very focal deletions is relatively straightforward, prioritizing many candidate genes encompassed by a single large deletion is more challenging. Focusing on the minimal common region (MCR) targeted by overlapping deletions can narrow the number of candidate genes, as can parallel gene expression analysis to identify the subset of genes within a deleted region that are expressed at lower levels [50]. In addition, choosing the subset of probes within an MCR with the greatest (or most significant) amplitude of copy number aberration has been used to further narrow the candidate list [51]. However, this requires that the probes overlying the targeted gene are also the most aberrant, an assumption that has been challenged by examining the characteristics of probes marking *bona fide *tumor suppressor genes [38]. A particularly novel approach to determining whether a large alteration may harbor more than one target is to remove (“peel-off”) the most aberrant (significant) subset of contiguous probes, and to recalculate the significance of the remaining probes and determine whether any other subset of contiguous probes still reaches significance [51,52]. 

## CANDIDATE NOVEL TUMOR SUPPRESSOR GENES IDENTIFIED BY COPY NUMBER ANALYSIS

Having reviewed the major steps involved in narrowing thousands of probe intensities to a manageable number of candidate tumor suppressor genes, we now discuss examples of specific candidate tumor suppressor genes identified on the basis of large-scale array-based copy number analyses. Although the list of candidate genes discussed is by no means comprehensive, our primary goal is illustrate how each candidate was selected and to critically evaluate the data subsequently obtained from deeper genetic and functional validation studies supporting a *bona fide *tumor suppressor function for each candidate. 

### Novel Glioma Tumor Suppressor Genes: *PARK2* and *PTPRD*

A comprehensive approach to converting probe-level intensity data to candidate cancer regions and genes has been utilized to characterize several cancer genomes, including those from gliomas, lung adenocarcinomas and renal cell carcinomas [51,52,54,70]. The approach, known as Genomic Identification of Significant Targets in Cancer, or GISTIC, illustrates many of the steps of analysis outlined in the first part of this review. First, a series of data preprocessing steps log2-transforms and median centers the probe intensity data (sample by sample), corrects for systematic variation in probe intensities apparent in subsets of arrays (“batch” effects), converts probe intensity to copy number by normalizing each tumor sample only to the subset of (unmatched) normal samples with similar overall probe behavior (“tangent” normalization), and removes duplicate samples and samples contaminated by significant stromal admixture. Next, probe-level copy number data is segmented (with the GLAD segmentation algorithm) and each segment is median smoothed (e.g. each probe is assigned a new copy number value equal to the median value for all probes contained in the same segment) [42]. Third, probes overlapping polymorphic regions as identified in the Database of Genomic Variants are removed [55] Fourth, each probe is weighted by the G-score, the product of aberration frequency and average amplitude across all samples, assigned significance by comparing the individual G-score to that obtained with an approximation of the null distribution, and the resulting p-values corrected for multiple comparisons to generate q-values. Probes with q-values with q-values <0.25 are considered to be significant. Finally, the candidate gene(s) is determined from the subset of probe(s) with the lowest q-values in each region.

Notable findings from GISTIC analysis with particular relevant to finding new TSGs include:

1. The background rate of deletions is higher than the background rate of amplifications. Therefore, deletions must occur at higher frequencies than amplifications to reach statistical significance (based on the G-score statistic described above). The background rate in gliomas is particularly high—every region of the genome is altered in at least one sample.
2. Both “broad” (at least a whole chromosome arm) and focal deletions are identified, with broad losses occurring substantially more frequently than focal losses.
3. In the glioma studies, the frequency of recurrence of focal alterations decreases with increasing size of the alteration, up until a size equal to ~90% of a chromosome arm, at which point the trend reverses. This could have important implications for the particular model used to estimate the background rate of alteration, since that rate may vary depending on whether the alteration is large or small.
4. Several *bona fide* glioma and lung adenocarcinoma tumor suppressor genes were identified, including *CDKN2A/B, PTEN, RB1 *and* CHD5 *(glioma) and *CDKN2A/B, TP53, STK11, PTEN *and* RB1 *(lung cancer).
5. Several deleted regions are not associated with known cancer related genes. Although the majority of these encompass many genes, the peak regions corresponding to several are limited to one or a few candidates.
6. The GISTIC method may be remarkably robust for a variety of studies and platforms. In the initial publication, when the data from two previous glioma studies (comprising different samples and array platforms) was reanalyzed with GISTIC, the candidate regions identified by each study were remarkably concordant, even though there was little overlap in the candidate regions when the original algorithms and prioritization schemes were utilized [71,72]. The combined analysis of samples from all three studies permitted adjustment of the peak regions in the previously identified intervals, and allowed eight aberrations (four gains and four losses) that fell above the initial q-value cutoff with the initial analysis to gain significance in the combined data set. Despite this, some regions in the initial study (e.g. encompassing *CDH5*) do not reach significance in the follow up study and vice versa (e.g. *NF1*, *CDKN2C*).
7. Resequencing of selected candidates in additional samples uncovered somatic mutations of *PTPRD *(see below), *CDKN2A, PTEN, RB1 *and* NF1*. Several of these are *bona fide *inactivating mutations (e.g. nonsense, frameshift, splice site), thus providing additional genetic evidence for somatic inactivation in human cancers.

Among the genes nominated as a novel candidate tumor suppressor gene by the glioma copy number analysis is *PARK2*. The gene encodes Parkin, an E3-ubiquitin ligase, mutations in which (including deletions) were shown by classical linkage analysis to account for the most common form of inherited juvenile Parkinson’s disease [73]. Loss-of-heterozygosity on the long arm of chromosome 6, centered on 6q25-27, has been frequently described in human tumors, and *PARK2 *(6q26) has previously been suggested to be a candidate tumor suppressor gene on the basis of heterozygous (and rare homozygous) deletions in diverse cancers [74-76]. However, the somatic origin of these mutations had not been demonstrated, and tumor re-sequencing initially failed to reveal inactivating point mutations.

The short arm of chromosome 6 was shown in both GISTIC glioma studies to harbor a highly significant recurrent region of loss. Although the number of contiguous probes significantly scoring as lost in these studies spans a large portion of 6q (essentially all of 6q scores as significant), the peak region defined by the set of contiguous probes with the smallest q-value contained only 3 genes: *PARK2, PACRG* and *QKI. *Based on these findings, Verriah *et al*. subsequently re-examined individual tumor sample copy number data from the TCGA set of 216 gliomas (as well as l98 colon cancer samples) [77]. These authors discovered loss of at least one allele of *PARK2 *in ~25% of each tumor type, with intragenic homozygous deletions within *PARK2 *in 2.3% gliomas (5 cases) and 6.1% colon carcinomas (6 cases). In addition, sequencing of 242 tumors identified 12 cases with point mutations (11 heterozygous), including a splice site mutation in a primary glioma and a nonsense mutation in a glioma cell line. Finally, the authors performed extensive functional validation in human cancer cell lines, demonstrating suppression of *in vitro *growth and colony formation and *in vivo *growth of cell line xenografts upon re-expression of wild-type but not several mutant *PARK2 *alleles, and only in cells with endogenous loss of *PARK2* (cells expressing wild-type *PARK2 *were not affected). Parkin had previously been shown to function as an E3 ubiquitin ligase, and the authors demonstrated wild-type but not cancer-specific mutant Parkin was able to direct the proteasomal-mediated degradation of Cyclin E, and that *PARK2* knockdown led to the an increased fraction of cells in the S and G2-M phases of the cell cycle. 

*	PTPRD*, encoding a receptor tyrosine phosphatase, is a second candidate tumor suppressor gene identified by GISTIC analysis. *PTPRD* was previously found to be deleted in both small cell and non-small cell lung cancer cell lines [78,79]. However, its proximity to *CDKN2A/B*, the high rate of homozygous deletions affecting 9p in lung cancer (~30%) and the identification of frequent germline copy number polymorphisms also raised the possibility that *PTPRD *is part of a fragile site prone to recombination [67,78] and is not a true tumor suppressor gene. Initial GISTIC analysis of 371 primary lung adenocarcinomas identified 5 tumors with focal deletions affecting the 5’ untranslated region of *PTPRD* [52]. Sequencing of all *PTPRD* coding exons in 188 tumors revealed somatic, heterozygous missense mutations in 11 samples (6%). Notably, sequencing of two other candidate genes encompassed by significant deletions (*AUTS2 *and* PDE4D*) for a total of 34 coding exons failed to uncover any mutations in these genes. Although *PTPRD *was not uniquely identified by the GISTIC analysis of gliomas, it was scored as one of the most significant candidate genes in the same data set using a different algorithm (RAE) [38]. Following up on these results, a subsequent re-analysis of the TCGA glioma data set revealed frequent (~40%) hemizygous or homozygous losses, most encompassing nearly all of chromosome 9p. The majority of these (two-thirds) demonstrated loss of either *PTPRD* or *CDKN2A/B*. Although *PTPRD *loss was accompanied by co-deletion of *CDKN2A/B* in all but one case, for one-third of co-deleted samples, a region of normal copy number separates the *PTPRD *and *CDKN2A/B *deletions (suggesting the possibility that each deletion was selected independently). Furthermore, two samples demonstrated intragenic homozygous deletions within the *PTPRD *gene that are predicted to remove coding exons. Additional evidence for somatic inactivation of *PTPRD *was provided both by sequencing analysis, which uncovered eight somatic mutations, including a single nonsense mutation, and by methylation-specific PCR and bisulfite sequencing, which uncovered specific methylation of the *PTPRD *promoter in ~ one-third of primary gliomas analyzed (as well as a significant fraction of breast and colon cancers). Finally, introduction of wild-type *PTPRD *(but not cancer-specific mutants) inhibited, and shRNA knockdown promoted, cell growth *in vitro* and in xenografts, possibly *via *effects on the phosphorylation-dependent activation of STAT3. 

For both *PARK2 *and *PTPRD, *the discovery of focal intragenic microdeletions, somatic point mutations (and for *PTPRD, *promoter methylation) and tumor suppressor activity in functional assays provide compelling evidence for their specific inactivation during gliomagenesis. However, two features of both of these genes complicate their classification as TSGs with a highly significant role in gliomagenesis. First, more than 100 germline genomic variants overlapping *PARK2 *and* PTPRD *have been identified, including 46 copy number polymorphisms (e.g. more than 1kB in size) in *PARK2 *and 40 in *PTPRD* (the remaining consist of small insertions and deletions from 0.1 to 1kB in size) [55]. For *PTPRD*, three losses overlap coding exons, one with a frequency of 3% (in the population examined). For *PARK2, *at least four losses eliminate coding exons, one at a frequency of ~5%. Although the initial genomic studies took steps to minimize the impact of germline polymorphisms on the selection of candidate regions (for the lung adenocarcinoma study in particular, matched normal samples were used to determine copy number for the majority of tumor samples), it is surprising that inactivating germline deletions occurring at a significant frequency in the general population are not associated with a highly penetrant predisposition to cancer (for example, compare this frequency to the much lower overall population frequency of germline inactivating mutations in *bona fide *tumor suppressor genes responsible for rare familial cancer predisposition syndromes). In addition, very few germline polymorphisms have been catalogued for established TSGs (Table **2**). This may be particularly relevant for *PARK2*, since most deletions are hemizygous, and the authors hypothesize that haploinsufficiency of *PARK2 *is sufficient to promote tumorigenesis. Second, although there is a precedent for heterozygous missense mutations leading to inactivation of protein function (e.g. the *TP53 *gene), generally this requires dominant-negative activity for the mutant protein or epigenetic loss of expression of the wild-type allele. In both studies, overexpressed cancer-specific mutant Parkin or PTPRD proteins lacked the growth inhibitory effects of the wild-type protein in cells with loss of the endogenous gene; however, there was no demonstration of potential dominant-negative activity by testing the effect of the mutants on cells wild-type for each protein. Furthermore, in tumors or cells with hemizygous mutations (deletions or point mutations), expression from the wild-type allele was not examined.

### The First X Chromosome Tumor Suppressor Genes: *WTX* and *UTX*

The discovery of *WTX *illustrates the power of genome-wide copy number analysis when focused on a limited set of highly related tumors with a low background rate of genomic alterations [19]. Wilms tumor is the most common pediatric kidney cancer. Germline heterozygous genetic inactivation of the zinc finger transcription factor *WT1 *accounts for two forms of familial Wilms tumor (the second allele is inactivated in the tumors), and somatic biallelic inactivation has been described in 5-10% of sporadic Wilms tumors [12,15,19]. In addition, activating mutations in the *CTNBB1* gene and epigenetic dysregulation of the IGF axis have been described in rare cases [80]. However, in the majority of cases, no specific genetic abnormalities had been identified.

To search for additional Wilms tumor genes, investigators performed oligonucleotide array-based CGH on a collection of 51 primary Wilms tumor specimens. In marked contrast to most adult cancers, the array CGH profile in Wilms tumor was remarkably stable, with very few gains, single copy losses or homozygous deletions. As a result, focal homozygous deletions at Xq11.1 spanning just 1-3 probes were able to be clearly identified in 5/26 tumors, with the minimal common area of deletion implicating a previously uncharacterized gene, which was renamed *WTX. *Sequencing of the coding region in additional tumors uncovered six truncating mutations; no mutations were present in matched normal tissue or 269 unmatched normal DNA samples. Notably, in Wilms tumors from female patients, inactivating mutations exclusively target the active X chromosome through a single hit mechanism, with expression of the intact allele eliminated by X inactivation. Furthermore, expression of *WTX* is restricted to the renal stem cell population that is the presumed cell of origin of Wilms tumors. Although cell lines with endogenous loss of *WTX* are not available, the authors showed that overexpression in immortalized kidney cells suppressed colony formation through induction of apoptosis. 

By comparison to the relatively focused analysis of a limited number of tumors of a single type, analysis of 1,390 cancer cell lines and primary tumors by a combination of array-based copy number analyses and candidate gene re-sequencing uncovered 39 inactivating mutations in the histone H3 lysine 27 demethylase gene *UTX*, also located on the X chromosome [81]*. *The mutations comprised 16 homozygous deletions (13 intragenic microdeletions) and 23 point mutations (nine nonsense, 12 frameshift and two splice site mutations). In contrast to *WTX, *mutations in which so far appear limited to Wilms tumors, *UTX* mutations were discovered in multiple tumor types. Furthermore, *UTX *has been shown to escape X inactivation in females. Instead, a Knudsen two-hit model of inactivation is likely, on the basis of specific loss of the second *UTX *allele in the majority of affected cell lines derived from cancers in females, and concurrent loss of the *UTX *Y chromosome paralogue *UTY *in cell lines derived from cancers in males. Finally, re-expression of wild-type *UTX *significantly prolonged the *in vitro *doubling time of two *UTX* mutant cell lines but not a wild-type cell line. Although no correlation was observed between *UTX *status and global H3K27 trimethylation, reintroduction of *UTX *resulted in decreased H3K27 trimethylation of differentially expressed genes. 

The combined genetic and functional data provide very compelling evidence for a *bona fide* tumor suppressor function for both *WTX* and *UTX. *In contrast to *PTPRD *and *PARK2,* highly frequent inactivating frameshifts and nonsense mutations were uncovered; no germline polymorphisms overlapping either* WTX *or *UTX *have previously been described; and no instances of inactivating mutations or deletions were identified in hundreds of normal DNA samples analyzed in each study. 

### Acute Lymphoblastic Leukemia (ALL) and *PAX5*

As with *WTX, *the discovery of inactivating mutations in *PAX5 *and other B-lymphocyte lineage determination genes in pediatric ALL illustrates the power of copy number analysis for uncovering critical tumor suppressor genes, particularly when focused on a single cancer type with a relatively low background rate of copy number alterations [82,83]. In contrast to Wilms tumor, tumor initiating genetic lesions in many pediatric ALLs have been defined, including chromosomal translocations (e.g. *TEL-AML1, E2A-PBX1, BCR-ABL, E2A-PBX1) *and rearrangements involving *MLL* [84]. However, none of these lesions yields a full leukemic phenotype in animal models, suggesting a requirement for additional cooperating alterations. To search for such genomic alterations, Mullighan *et al.* interrogated the purified leukemic blasts from 242 cases of pediatric ALL using high-resolution SNP arrays. Copy number was determined in a novel fashion, by normalizing each probe to the intensity of probes derived from known diploid regions within the same sample’s genome (as determined by cytogenetics) prior to segmentation. Segments comprised of less than 3 SNPs were filtered out, and the remaining segments that contained an average log2 copy number ratio ≥ 0.2 or ≤ -0.2 were scored as gains or losses. Germline copy number variants were eliminated for 228 samples by using the available paired remission sample as the copy number reference, and for the 14 unmatched samples by eliminating probes that appeared polymorphic in the original pool of reference samples. 

Notably, the mean number of copy number alterations per tumor is only 6.46, with amplifications outnumbering deletions ~2:1 (this is the opposite of what was observed for gliomas). Among 54 recurrently deleted regions were previously identified *bona fide *tumor suppressor genes in leukemias, including *CDKN2A, ATM, *and* RB1. *However, most strikingly were 91 deletions in 7 regulators of normal B-lymphocyte development, limited to B-lymphocyte-derived ALL cases (n=192). Among these were 57 tumors with deletions of *PAX5 *(53 single copy, 3 biallelic, 1 internal amplification, 23 intragenic), resulting in loss of expression of the mutated allele or expression of internally deleted or truncated proteins lacking critical functional domains. Importantly, since the majority of *PAX5 *mutations were heterozygous, the authors tested the functional effects of a panel of cancer-specific mutant *PAX5 *target genes in the presence of the wild-type protein. In all cases, the mutant proteins inhibited transcriptional activation of transfected reporter constructs and known endogenous *PAX5 *target genes. Using a similar analysis, the same authors recently demonstrated recurrent intragenic deletions in the B-lineage developmental regulator *IKZF1 *in more than 80% of *BCR-ABL *positive ALL cases [83]. The high frequency of genetic inactivation of several genes comprising the same pathway (e.g. B lineage determination), the demonstration of dominant-negative activity, the lack of previously described germline variants and the use of matched normal reference samples to confirm the somatic origin of the observed mutations provide strong evidence for a highly frequent and penetrant tumor suppressor role for these genes. 

## CONCLUSIONS

In no other field of biology or medicine has the potential value of genome-wide approaches been as thoroughly exploited as human cancer genetics. An unprecedented body of data describing human tumor copy number aberrations has been accumulated in recent years, leading to the identification of many recurrent regions of gains and losses that may harbor critical cancer genes. These studies have been carried out on a variety of array platforms, using a variety of algorithms for identifying genomic regions, different thresholds for scoring gains and losses and various criteria for prioritizing candidate genes and determining statistical significance. As discussed, given the particular challenges associated with detecting genomic losses (compared to gains), accurate detection of single-copy and homozygous deletions may depend on the specific assumptions and methodologies of the individual approaches. Therefore, knowledge of how each method specifically manages the detection of losses can be helpful in choosing individual candidates for further validation from large lists of genes.

When evaluating the evidence supporting a tumor suppressor role for a candidate gene identified through array-based copy number analysis, several critical questions need to be considered. Which array platform was used? What was the density of probes? What was the nature of the reference samples used to determine copy number? If segmentation was used, how were very small segments considered? What types of thresholds were used to select losses? Were germline copy number variants considered? Was the background rate of losses estimated (and how)? Were individual candidates genes validated with deeper analysis and functional assays? Do the mutations (deletions, point mutations) disrupt both alleles? If not, is the wild-type allele expressed? 

The shear volume of cancer genomic data will continue to increase as newer technologies, such as whole cancer genome re-sequencing and genome-wide analysis of epigenetic DNA modifications, are refined to permit their application to large numbers of samples. This overview of array-based copy number analysis hopefully provides a framework for evaluating the large number of candidate tumor suppressor genes likely to emerge from these technologies in the near future.

## Figures and Tables

**Fig. (2):**
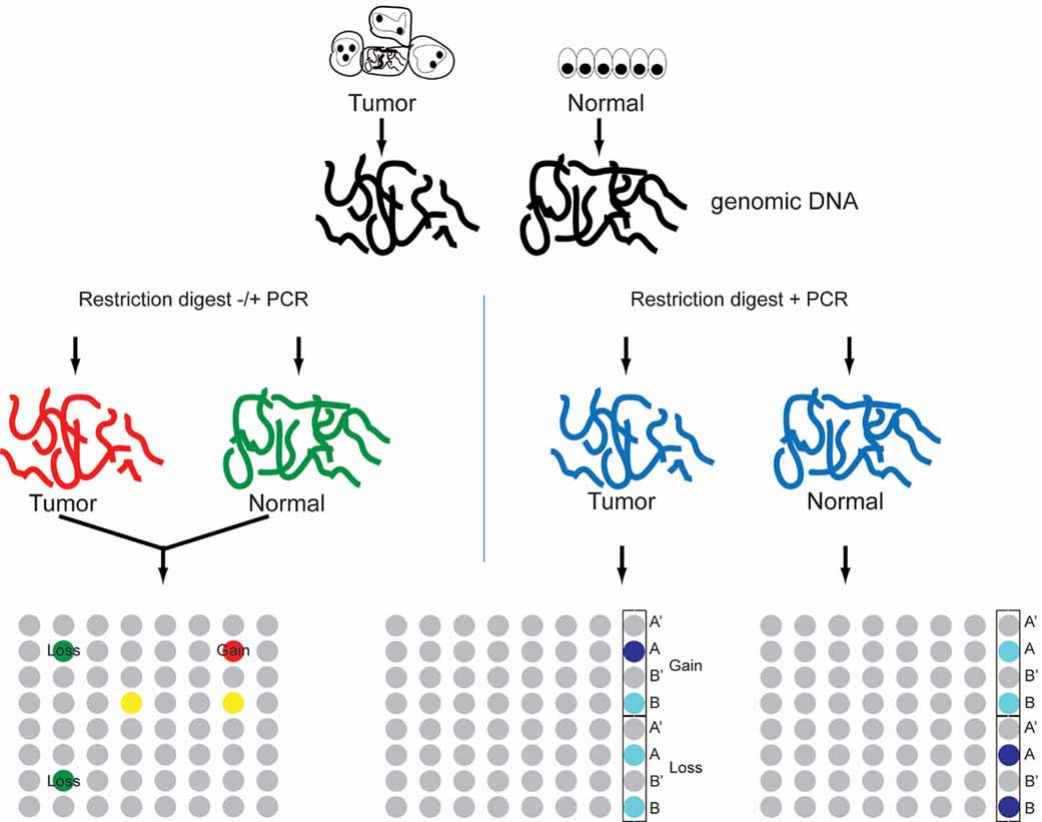
Comparison of major copy number analysis platforms. With traditional array CGH (left), genomic DNA isolated from tumor and normal samples is labeled with unique fluorophores (e.g. Cy5 (red) for tumor and Cy3 (green) for normal and then cohybridized to a single array consisting of cDNAs or 60-70mer oligonucleotides. Genomic losses are identified as an abundance of Cy3 (green) fluorescence, gains as an excess of Cy5 (red) fluorescence. With SNP arrays (right), tumor and normal samples are labeled and hybridized in parallel to different arrays. Each SNP probe consists of four sets of 25mer oligonucleotides, two each for the major (**A**) and minor (**B**) alleles, one perfectly matched (**A,B**) and one mismatched (**A',B'**). Copy number is determined *in silico* by accounting for the total hybridization signal from eachperfectly matched probe, normalized to the background hybridization from the mismatched probes and the average hybridization signal fromthe normal samples. An abundance of signal (increasing blue) in normal relative to tumor marks genomic losses, in tumor relative to normal,gains.

**Table 1.** Examples of *Bona fide* Tumor Suppressor Genes Identified by Focal Deletions

Gene	Methodology	Size of minimal deletion	Reference
*CDKN2A/B*	Deletion mapping	intragenic	[17,22]
*Rb1*	Linkage^*^	intragenic	[13]
*APC*	Linkage	three genes	[14,16]
*WT1*	Linkage	25 nucleotides	[12,15]
*SMAD4*	LOH	2Mb	[21]
*PTEN*	RDA, LOH	intragenic	[18,20]
*WTX*	array CGH	1-3 oligonucleotide probes	[19]

* Homozygous deletions in sporadic tumors.

**Table 2.** Frequency of Germline Genomic Variants for *Bona fide* and Putative Tumor Suppressor Genes

Candidate	Cytoband	CNV	INDEL
*MSH2*	2p21	0	1^*^
*TGFBR2*	3p24.1	0	1^*^
*CDKN2A/B*	9p21.3	1	0
*PTEN*	10q23.31	0	0
*WT1*	11p13	1	0
*RB1*	13q14.2	0	2^*^
*TP53*	17p13.1	0	0
*NF1*	17q11.2	0	0
*SMAD4*	18q21.1	0	0
*NF2*	22q12.1	0	0
*WTX*	Xq11.1	0	0
*FHIT*	3p14.2	16	4
*PARK2*	6q26	35	10
*CSMD1*	8p23.2	60	31
*PTPRD*	9p23	30	19
*WWOX*	16q23.1	18	3

CNV: copy number; INDEL: insertion-deletion.

* intronic.

Source: Database of Genomic Variants [55].
